# Sodium Lignosulfonate Modified Polystyrene for the Removal of Phenol from Wastewater

**DOI:** 10.3390/polym12112496

**Published:** 2020-10-27

**Authors:** Keyan Yang, Jingchen Xing, Jianmin Chang, Fei Gu, Zheng Li, Zhenhua Huang, Liping Cai

**Affiliations:** 1 College of Material Science and Technology, Beijing Forestry University, Beijing 100083, China; ykysdut@163.com (K.Y.); jingchenx612@163.com (J.X.); 15922213914@163.com (F.G.); li15127802822@163.com (Z.L.); 2 Department of Mechanical Engineering, University of North Texas, Denton, TX 76207, USA; zhenhua.huang@unt.edu (Z.H.); liping.cai@unt.edu (L.C.); 3 College of Materials Science and Engineering, Nanjing Forestry University, Nanjing 210037, China

**Keywords:** sodium lignosulfonate, polystyrene, phenol, adsorption

## Abstract

An eco-friendly and novel water treatment material was synthesized using sodium lignosulfonate modified polystyrene (SLPS), which can be used to eliminate phenols in aqueous solution. SLPS was characterized by BET, FTIR, SEM, and EDS. The effect of the initial pH value, phenol content, adsorption time, and temperature on the absorbability of phenol in SLPS was investigated through adsorption experiments. It was found that SLPS could efficiently adsorb phenol in aqueous solution at a pH value of about 7. The test results revealed that the kinetic adsorption and isotherm adsorption could be successfully described using the pseudo second-order and Langmuir models, respectively. It was illustrated that the phenol adsorption on SLPS was dominated by chemisorption and belonged to monolayer adsorption. The max. phenol adsorption value of SLPS was 31.08 mg/g at 30 °C. Therefore, SLPS displayed a great potential for eliminating phenol from polluted water as a kind of novel and effective adsorbent.

## 1. Introduction

Phenol and phenolic compounds are important organic intermediate chemicals obtained from industrial processes, and are widely used in the industrial production of petrochemicals, oil refining, pesticides, disinfectors, and synthetic resin [1,2,3,4,5,6]. Phenol and its derivatives inevitably flow into natural water ecosystems with industrial wastewater and become a kind of harmful environmental contaminant, which has resulted in severe environmental pollution over the past few decades [7]. According to the Environmental Protection Agency of the US and National Pollutant Release Inventory of Canada, phenol is considered to be one of the most important pollutants [8,9,10]. There are strict discharge limits for phenol. The maximum phenol concentration is 0.1 mg/L in wastewater and the permissible content of phenol is less than 1 μg/L in drinking water [11,12,13]. Phenol can harm human health, potentially causing acute and chronic diseases [14]. Human contact with large doses of phenol can cause serious muscle fatigue, skin rashes, and diarrhea [14,15,16]. Phenol even causes bronchoconstriction, adverse effects in the lungs, suicidal death in red blood cells, and comas at lethal doses in humans [17,18,19,20]. It can also be inhaled or absorbed through the skin and digestive system, even at very low doses, in water [21]. Chronic exposure to phenol can harm the liver, kidney, central nervous system, and other organs [22]. Therefore, it is necessary to remove phenol from industrial wastewater before discharging it, in order to protect the water ecological environment and sustain human physical health.

Many techniques, such as catalytic oxidation [23,24,25], membrane filtration [26,27], coagulation [28], ion exchange [29], solvent extraction [30], and adsorption [31], have been investigated to remove phenol from aquatic environments. The adsorption method by adsorbent has been proven to be a preferred method for eliminating phenol from polluted water because of its low operating cost, large adsorption capacity, and non-hazardous technique compared to the other methods [9,31]. The adsorption process has been widely applied for various contaminants, such as heavy metal ions, dyestuff, pesticides, and phenol. There are many types of adsorbing materials that have been developed for wastewater treatments, such as activated carbon [32,33], resins [21], ion exchange resin [14], turf soil [8], clarified sludge [34], and zeolites [35]. As a low-cost adsorbent with a porous structure, polystyrene has been increasingly used in the adsorption of pollutants such as phenolic compounds, oil, drugs, perfluoroalkyl acids, triazole fungicides, catecholamines, etc. [36,37,38,39,40,41]. The surface features and porous nature of polystyrene are important factors affecting phenol’s adsorption performance. The undeveloped capillary structure of polystyrene can slightly promote its adsorption efficacy and the adsorption of polystyrene for pollution is limited, due to the lack of active adsorption sites [21,42]. Polystyrene is not used as a good adsorption platform for phenol removal in current industrial applications. Previous studies have found that phenol adsorption of adsorbents can be promoted with aromatic rings and functional groups, e.g., carboxyl and carbonyl [35]. Therefore, the adsorption performance of polystyrene for phenol can be improved by surface functional group modification. A novel, cost-effective, and efficient material with aromatic rings and functional groups is highly needed. Sodium lignosulfonate is a kind of natural polymer with abundant reserves and environmental friendliness, and is renewable and low cost. As an inevitable by-product, the paper and pulp industry is an important source of sodium lignosulfonate. Although sodium lignosulfonate is abundant in reserves, its utilization rate is not high at the present time. Unreasonable ways of dealing with sodium lignosulfonate, such as dumping it in land-fill places or rivers, exert more pressure on the environment and waste useful resources. Some researchers have also found that sodium lignosulfonate and its derivatives have hydroxyl, hydroxymethyl, sulfonate, ether bond, carboxyl, carbonyl, and many other oxygen-containing functional groups. These active functional groups greatly improved its chemical reactivity and water solubility [43,44]. At the same time, sodium lignosulfonate and its derivatives have been shown to adsorb pollutants, such as phenol and heavy metals [45,46]. Sodium lignosulfonate can be used as a modifier to improve the adsorption performance of polystyrene to phenol due to its large number of oxygen-containing functional groups. Therefore, the preparation of adsorbents with polystyrene modified by sodium lignosulfonate to remove phenol from polluted aquatic environments is feasible for realizing the high-value utilization of sodium lignosulfonate, as well as improving the adsorption properties of polystyrene.

In this paper, the wastewater treatment material was synthesized using sodium lignosulfonate modified polystyrene (SLPS), which can be used for removing phenol in aqueous solution. The porous structure surface, morphology, and chemical composition of SLPS were characterized. The effect of the initial pH value, phenol content, adsorption time, and temperature on the absorbability of phenol in SLPS was studied by adsorption experiments. The adsorption mechanism was explained, and the potential was evaluated by measuring the SLPS removal of phenol from polluted water using adsorption kinetic and isotherm models.

## 2. Material and Methods

### 2.1. Material

Chloromethyl polystyrene (CPS) was supplied by Tianjin XingNan Technology Co., Ltd. (Tianjin, China). The CPS was prepared by a chloromethylation reaction with polystyrene and chloromethyl methyl ether as starting materials and zinc chloride as condensing agent. The CPS had a crosslinking degree of 7%, chlorine content of 17%, and average particle diameter of 0.3–1.25 mm. Obtained from Macklin Chemicals Co., Ltd. (Shanghai, China), the sodium lignosulfonate mainly contained 28.32% oxygen, 41.63% carbon, and 24.39% sodium, as well as a small amount of sulfur and silicon. 1,3-diaminopropane (C_3_H_10_N_2_), tetrahydrofuran, and phenol were purchased from Macklin Chemical Co., Ltd. (Shanghai, China). Formaldehyde (HCHO) was attained from Xilong Scientific Co., Ltd. (Shantou, China). All chemicals used in the experiments were of analytical grades.

### 2.2. Preparation of SLPS

**Figure 1:**
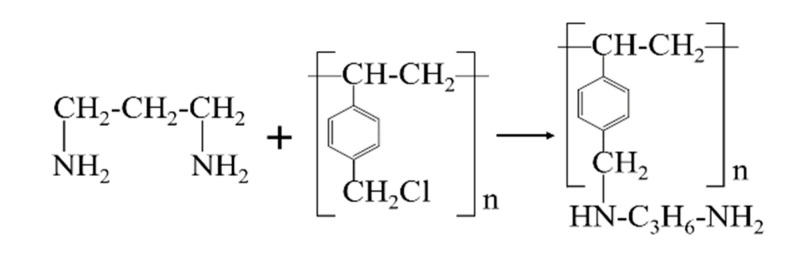
The amination mechanism of ACPS.

There were two main steps employed for the preparation of SLPS, including amination pretreatment of CPS and a Mannich-type reaction with sodium lignosulfonate and ACPS (aminated chloromethyl polystyrene). The CPS was amination-treated with 1,3-diaminopropane through an amination reaction. The amination mechanism of ACPS is shown in Figure 1. At first, 5 g CPS was swollen for 2 h with 15 mL tetrahydrofuran in an Erlenmeyer flask. Then, 25 mL of 1,3-diaminopropane was added to the flask. The swollen CPS amination reacted for 12 h in the 50 °C water bath with 1,3-diaminopropane. The reaction product was filtered out of the solution and dried at 105 °C for 6 h, which was named ACPS.

**Figure 2:**
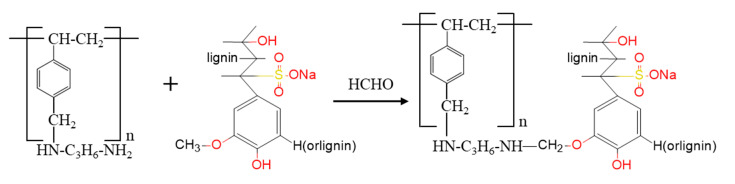
The reaction scheme of sodium lignosulfonate modified polystyrene (SLPS).

The SLPS was prepared by using the sodium lignosulfonate and ACPS as raw materials. The reaction mechanism of SLPS is shown in Figure 2. A total of 5 g ACPS was swollen for 2 h with 15 mL tetrahydrofuran in an Erlenmeyer flask. Next, 40 mL 20% formaldehyde solution was added to the flask and 5 g sodium lignosulfonate was then added to the system after 30 min. Following this, sodium lignosulfonate reacted with ACPS in the 90 °C water bath for 12 h. After the reaction with room-temperature cooling, the flask was removed from the water bath. The resulting products were successively filtered, washed two times with the diluted hydrochloric acid (0.5 mol/L), and then washed with distilled water until they were neutral. The sample of SLPS was obtained by drying for 6 h at 105 °C.

### 2.3. Characterizations

The surface BET value, and the volume and size of pores were determined using the physisorption analyzer (autosorb-IQ, Quantachrome, Boynton Beach, FL, USA) by N_2_ adsorption isotherms at 77 K, following the BET method (Brunauer-Emmett-Teller method). The surface morphologies of CPS and SLPS were observed by scanning electron microscopy (SEM, Hitachi S4800, Tokyo, Japan), and the microzone elements and distribution were characterized by dispersive spectrometry (EDS) and elemental mapping. The compositions of the carbon, oxygen, and sodium of sodium lignosulfonate were determined by an Elementar Analyzer (5E Series, Micromeritics Co., Norcross, GA, USA). A Fourier transform infrared spectra (FT-IR) spectrometer (iS10, Thermo Fisher Scientific, San Jose, CA, USA) was used to analyze the samples’ surface functional groups.

### 2.4. Adsorption Experiment

In the adsorption study, the absorbance of phenol solution with different known concentrations was firstly performed at a wavelength of 270 nm (isosbestic point) by a UV-Vis spectrophotometer [13]. A regression equation fitted with the absorbency and different known phenol concentrations was obtained with the correlation coefficient *R*^2^ of 0.9998. All batch experiments were performed for the measurement of phenol adsorption by taking SLPS as the adsorbent in the conical flasks. The experiments were conducted using 100 mg SLPS in 100 mL phenol solution, in order to investigate the influence of different solution initial pH values, contact times, temperatures, and initial pollutant concentrations on the absorbability of SLPS for phenol. The conical flasks with SLPS and phenol solution were then placed on a shaking table and shaken at 150 rpm. The filtrate was filtered out by a 0.45 μm filter after the adsorption and was determined as absorbance using the UV-Vis spectrophotometer, and the phenol concentration of filtrate was calculated according to the fitted regression equation. The effects of the initial pH value of 1.0 to 9.0 of the solution system on the absorbability of SLPS for phenol was studied. The initial pH of the system was adjusted by 0.1 mol/L HCl solution and 0.1 mol/L NaOH solution and the contact time was 6 h in the experiments. The effect of time on the absorbability of SLPS for phenol was studied by adsorption experiments using contact times ranging from 1 min to 24 h. The results were fitting by adsorption kinetics to explain the adsorption performance. Adsorption isotherms of SLPS for phenol at 20, 30, and 40 °C were tested using initial phenol concentrations ranging from 50 to 500 mg/L, respectively.

The absorbability *q_e_* of phenol onto SLPS was calculated using Equation (1) [33]:

(1) $$q_{e}=\frac{\left(C_{i}-C_{e}\right)V}{M},$$

where *q_e_* (mg/g) is the adsorption efficiency, $C_{i}$ (mg/L) is the initial phenol content, $C_{e}$ (mg/L) is the phenol content of filtrate, V (L) is the volume of phenol solution, and M(g) is the mass of SLPS.

## 3. Results and Discussion

### 3.1. Characterizations

The porous structure parameters of sodium lignosulfonate (SL), CPS, and SLPS are shown in Table 1. All samples exhibited a bad porous structure, especially sodium lignosulfonate. The specific surface area and pore volume parameters were almost zero and the average pore diameter of 11.75 nm was detected. SLPS had smaller specific surface area and pore volume and larger average pore diameter than CPS. This was because the reaction between the sodium lignosulfonate and ACPS caused micropores to be clogged in CPS, making the average pore size increase to 66.99 nm of SLPS from 45.38 nm of CPS. Although SLPS had a lower pore volume and surface area, its larger average pore size was obvious and beneficial for allowing the phenol molecules to enter the internal area of SLPS. It displayed a great potential for replacing traditional adsorbents.

**Figure 3:**
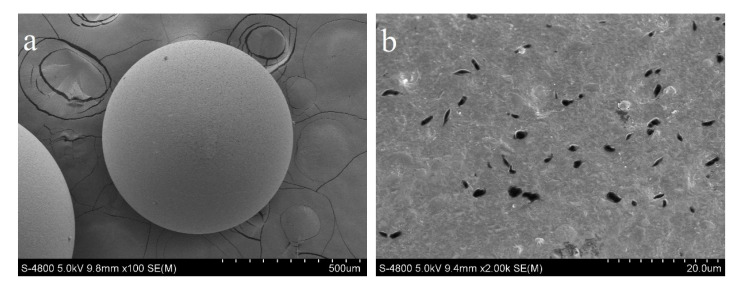

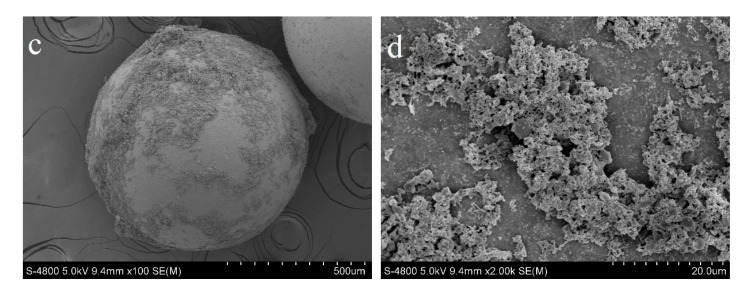
SEM images of the microsphere surface of chloromethyl polystyrene (CPS) (**a**,**b**) and SLPS (**c**,**d**).

**Figure 4:**
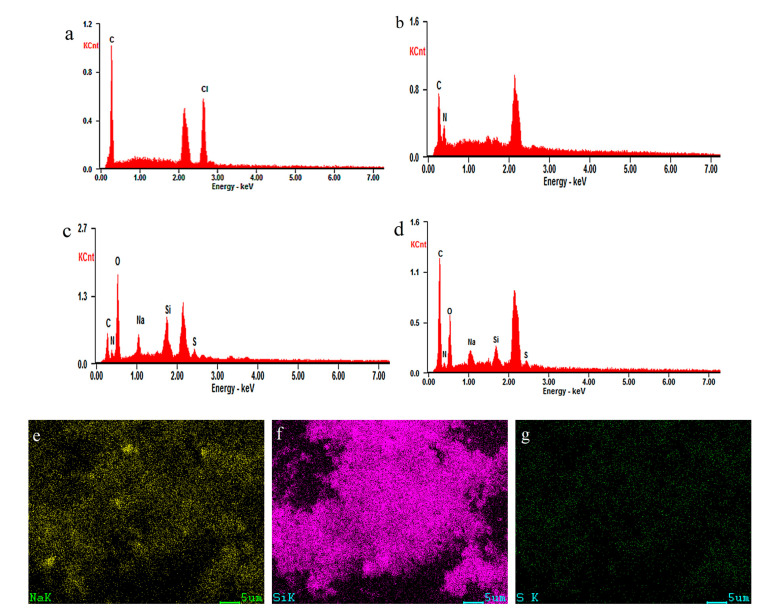
EDS spectrum of the CPS, ACPS, SLPS, and SLPS-P (**a**–**d**) and elemental mapping of Na, Si, and S of SLPS (**e**–**g**).

The microstructure of CPS and SLPS was characterized by SEM. The surfaces with different values of CPS are presented in Figure 3a,b and SLPS are presented in Figure 3c,d. As shown in Figure 3c, most SLPS particles were regular spheres with a diameter of about 1 mm. Due to the uniform macroscopic dimensions, SLPS can be filtered out very easily from solution after adsorption using the filtering method. There were numerous clearances and cracks with different sizes on the SLPS surfaces. The pore structure of adsorbent was the main channel through which the adsorbate entered the inside and was a necessary condition for physical adsorption. However, the BET parameters of SLPS indicated that the porous structure was not well-developed, which might have a negative impact on the physical adsorption of SLPS. Figure 3 illustrates that tiny pores were plugged and a graft appeared on surfaces of SLPS compared with CPS. This meant that there was a good reaction between sodium lignosulfonate and CPS through which the sodium lignosulfonate was modified on polystyrene. The EDS spectrum of the CPS, ACPS, SLPS, and SLPS-P (the SLPS after phenol adsorption) samples and the surface microstructure elemental mapping patterns of SLPS are displayed in Figure 4. It was found that the major elements on the CPS surface were C and Cl, and the Cl of ACPS disappeared, but nitrogen appeared, after the amination of CPS. The disappearance of Cl means that the replacement with diaminopropane and nitrogenous functional groups was introduced to ACPS. The Na, S, O, and Si appeared in the EDS spectrum of SLPS compared to CPS, among which the N content relative to the S content of the SLPS was 9.23. The elements both came from the raw material sodium lignosulfonate. The EDS mapping indicated that the SLPS surface was uniformly covered by Na, Si, and S. The O content of SLPS-P decreased, the C content increased, and the ratio of Na to S increased from 6.05 to 7.89 after phenol adsorption. It was shown that, after the polymerization of sodium lignosulfonate to CPS, the sulfo groups on the lignosulfonate had an active adsorption effect on phenol. The adsorption effect should be pH-dependent, due to the acid centers in the adsorbent.

**Figure 5:**
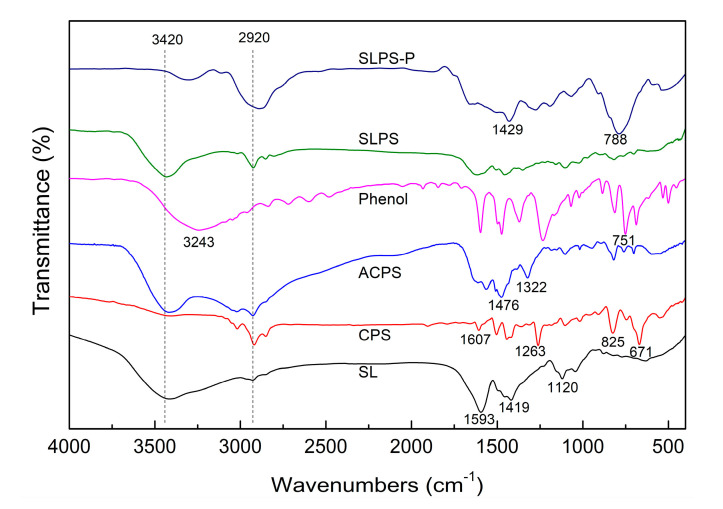
FT-IR spectra of the sodium lignosulfonate (SL), CPS, ACPS, phenol, SLPS, and SLPS-P samples.

The structural variation of the sodium lignosulfonate, CPS, ACPS, phenol, SLPS, and SLPS-P samples was examined by FTIR and the resulting spectra are illustrated in Figure 5. As can be seen from the figure, there is a broad peak of spectra at 3420 cm^−1^. This was caused by the stretching vibrations of the alcohols and phenolic hydroxyl groups associated with hydrogen bonds in the molecules. The adsorption peaks at 1593 and 1419 cm^−1^ of the sodium lignosulfonate structure can be attributed to the skeleton vibration caused by the stretching vibration of carbon atoms in the benzene ring. It was indicated that there may be many binary substituents on the benzene ring of sodium lignosulfonate. The adsorption peaks at 1120 cm^−1^ of the sodium lignosulfonate structure could have been caused by the stretching vibration of C–O, C–S, and other single bonds without hydrogen or S=O and other double bonds with heavy atoms. The FTIR spectrum curve of sodium lignosulfonate and CPS displayed small adsorption peaks at 2920 and 2850 cm^−1^ because of CH_2_ vibration. There were many less obvious sharp peaks at 1607 to 1263 cm^−1^ for CPS. These were caused by the stretching vibration of incomplete polymerized C=C and the bending vibration of methyl or methylene in olefin. The adsorption peaks at 671 and 825 cm^−1^ of the IR fingerprint spectrum were caused by C–H out-of-plane bending vibration on the CPS benzene ring. The ACPS after amination treatment showed an adsorption peak at 3500 to 3300 cm^−1^ and this peak belonged to the N–H stretching vibration band. The shape of this peak was much sharper than the hydroxyl group peak of sodium lignosulfonate and was a symmetric unimodal peak. This was likely due to the amino compounds of ACPS mostly existing as secondary amine. It is worth noting that the peaks’ intensity of ACPS was significantly stronger compared with CPS. This indicated that a lot of methylene was introduced to ACPS. The appearance of secondary amine and a lot of methylene in the FTIR spectrum of ACPS showed the CPS produced good amination modification by 1,3-diaminopropane. After the modification, the –OH and amine peaks rapidly decreased in prepared SLPS and the methylene adsorption peak disappeared, which meant that sodium lignosulfonate combined with ACPS and formed a new material. The broad peaks at 3420 and 2920 cm^−1^ of SLPS-P shifted to the right after phenol adsorption and peaks at 1429 and 788 cm^−1^ appeared, which indicated that phenol was successfully adsorbed by SLPS and attached onto its surface and the adsorption was governed by chemisorption.

### 3.2. Effect of the Initial pH Value

**Figure 6:**
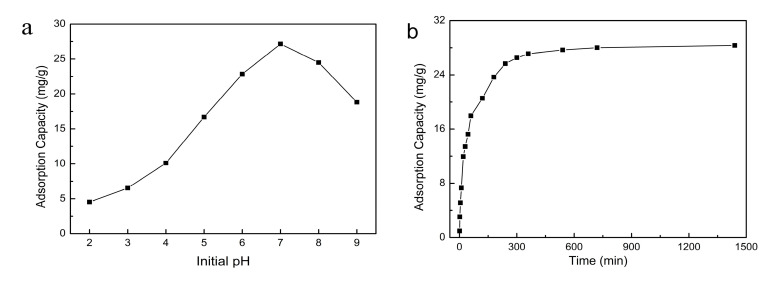
(**a**) Effect of the initial pH on the absorbability of SLPS for phenol and (**b**) effect of time on the absorbability of SLPS for phenol.

The existing form of adsorbate and the absorbability of adsorbent are different for different initial pH values of the solution system. The influence of the initial pH value of solution on the absorbability of SLPS for phenol was investigated in this research, and the influence trend in a line chart is shown in Figure 6a. The tests revealed that the absorbability of SLPS was firstly strengthened and then weakened when the solution’s initial pH increased from 2.0 to 9.0. The influence of the initial pH was identical to the results of other adsorption resins reported [14,21]. The smaller the pH value was, the stronger the acidity of solution and the higher the concentration of hydrogen ions in the solution. The hydrogen ions might induce a cation exchange reaction with SLPS to occupy adsorption sites and result in degradation of the adsorption property. In addition, phenol mainly existed in the form of phenol molecules, which were difficult to absorb while the solution was acidic. A high content of hydrogen ions was beneficial for encouraging phenoxy ions to lose electrons and form phenoxy radicals. However, the electrostatic adsorption between phenoxy radicals and SLPS was far lower than for phenoxy ions. Therefore, the absorbability of SLPS for phenol was very small when the solution’s initial pH value was low. The hydrogen bonding was also an important factor affecting the adsorption [21]. The existing form of phenol was gradually transformed from a phenol molecule to a phenoxy ion with the increase of the pH value, and the oxidation of a hydrogen ion to a phenoxy ion was weakened at the same time. The negatively charged phenoxy ion was combined with the positively charged adsorbent and there was a strong electrostatic interaction between the phenol and SLPS. The adsorption greatly benefited from the robust electrostatic interaction. The solution gradually turned to alkaline from acidic and the content of free hydroxyl ions in the solution was increased with the continuing increase of the pH value. The hydroxyl ions were in competition with phenoxy ions for the binding sites on the SLPS surface because they were both negatively charged. Therefore, the higher the pH values, the higher the content of hydroxyl ions, and the stronger the competitive adsorption. Both electrostatic repulsion and competitive adsorption had certain unfavorable effects on the adsorption. These results indicated that the initial pH of solution was an important factor influencing the absorbability and a pH value of 7.0 could help the adsorption of SLPS for phenol.

### 3.3. Kinetics of Adsorption

The change of the adsorption value of SLPS for phenol over time is shown in Figure 6b, which revealed that the phenol adsorption value increased rapidly in the initial stage of adsorption, especially in the first 3 h. Then, the adsorption rate gradually slowed down at 3 to 6 h after the start of adsorption, and the phenol adsorption value was 27.16 mg/g at 6 h. Next, the adsorption velocity further decreased, and adsorption gradually reached equilibrium with time. The adsorption of SLPS for phenol was a dynamic process with a gradually decreasing adsorption rate. The kinetic behavior from 1 to 1440 min (24 h) was analyzed for evaluating the adsorption process. The kinetics of adsorption was significant in confirming the adsorption type and explaining the adsorption process [34]. The test data of SLPS adsorption for phenol over time were fitted with pseudo first-order (PFO), pseudo second-order (PSO), and Elovich equations, in order to understand the controlling mechanisms.

These equations are shown as follows.

(2) $$\mathrm{PFO}:\;q_{t}\;=\;q_{e}\left(1-e^{-k_{1}t}\right),$$

(3) $$\mathrm{PSO}:\;q_{t}\;=\;\frac{q_{e}^{2}k_{2}t}{1+q_{e}k_{2}t},$$

(4) $$\mathrm{Elovich}:\;q_{t}\;=\;\frac{1}{\beta }\ln\left(1+\alpha \beta t\right),$$

where $q_{t}$ (mg/g) is the absorbability at time *t*, $q_{e}$ (mg/g) is the equilibrium absorbability, t (min) is the adsorption time, $k_{1}$ (min^−1^) is the PFO constant, $k_{2}$ (g/mg·min) is the PSO constant, and α and β are the Elovich constants.

The dynamic fitting parameters of different adsorption kinetic models are summarized in Table 2. As can be seen in the fitted results, the determination coefficient of the PSO model was larger than that of PFO and Elovich models. The PSO model matched better with the experimental results than others for describing the adsorption of phenol onto SLPS, indicating that the adsorption was dominated by chemisorption. There could be several paths for the adsorption process of SLPS: The phenol in solution firstly diffused onto the adsorbent surface and bound; pollutants entered the adsorbent through pores; and the adsorption occurred on the internal surfaces of the adsorbent. During the initial adsorption process, the pore structure was not completely filled and available active sites on the surface were sufficient to bind with pollutants. Since enough active sites of the SLPS surface were available, the mass transfer resistance of phenol was small. In addition, a high phenol concentration provided a greater driving force to contact active sites and be adsorbed. With the extension of time, the active sorption sites on the SLPS surface were gradually occupied and the concentration of phenol in the solution decreased. Some of the phenol entered the SLPS through the pores and was adsorbed by internal active sites or the internal pore structure. As the mass transfer resistance increased, the adsorption process became more difficult, leading to a decrease in the adsorption rate. When the active sites on the inner and outer surfaces of SLPS were completely occupied, the absorbability reached saturation, and the adsorption of SLPS for phenol gradually reached equilibrium.

### 3.4. Adsorption Isotherm

**Figure 7:**
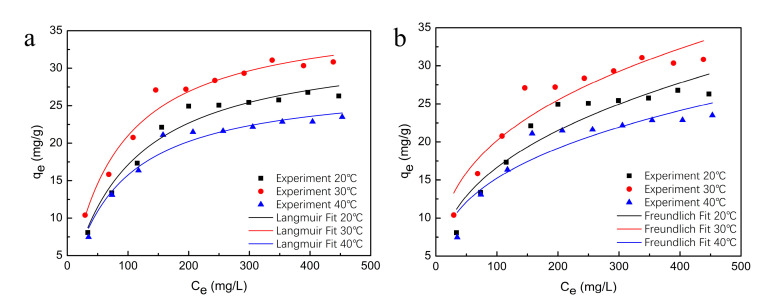
Adsorption equilibrium with different initial phenol concentrations and (**a**) Langmuir isotherm model fitting curves, and (**b**) Freundlich isotherm model fitting curves.

The influence of the initial phenol content on the SLPS adsorption was investigated according to the batch experiments with different phenol contents in the solution (50–500 mg/L). The test results of the equilibrium phenol concentration and the maximum absorbability are shown in Figure 7. The equilibrium phenol concentration increased and the equilibrium adsorption value of SLPS for phenol went up quickly and then slowly, with the increase of the initial phenol concentration. This may be because the higher the initial phenol concentration was, the more phenol there was in the filtrate, so the higher the equilibrium concentration was after adsorption was reached. In this case, the smaller the mass transfer resistance of phenol was, the stronger the equilibrium absorbability of SLPS for phenol was. The adsorption fluctuation of phenol was small with the initial phenol content increasing and the absorbability of SLPS reached the upper limit when all adsorption active sites were occupied.

Isothermal adsorption refers to the curve, in which the absorbability changes with the equilibrium content of pollution under the condition of a constant temperature and equilibrium adsorption, which can be used to evaluate the adsorption type of pollutant adsorbed by an adsorbent [34]. The Langmuir and Freundlich models were utilized for fitting equilibrium test data of adsorption at different temperatures.

These two isotherm equations are as follows:

(5) $$\mathrm{Langmuir}:\;q_{e}\;=\;\frac{K_{L}C_{e}q_{m}}{1+K_{L}C_{e}},$$

(6) $$R_{L}\;=\;\frac{1}{1+K_{L}C_{0}},$$

(7) $$\mathrm{Freundlich}:\;q_{e}\;=\;K_{F}C_{e}^{\frac{1}{n}},$$

where $q_{e}$ (mg/g) is the max. absorbability, $C_{e}$ (mg/L) is the equilibrium phenol content, $q_{m}$ (mg/g) is the monolayer absorbability, $K_{L}$ (L/mg) is the Langmuir constant, $R_{L}$ (L/mg) is the separation factor, $C_{0}$ (mg/L) is the initial phenol concentration $,\;\mathrm{and}\;K_{F}$ (L/mg) and n are the Freundlich constants.

Figure 7 shows the experimental data of adsorption at 20, 30, and 40 ℃ and non-linear fitting curves of Langmuir and Freundlich isotherm models, and Table 3 lists the fitting parameters of both models. The value of determination coefficient of the Langmuir model was larger than that of the Freundlich model, indicating that it agreed with the description of the adsorption type of SLPS for phenol. The adsorption type of SLPS for phenol belonged to monolayer adsorption. It can be concluded that the chances of phenol molecules occupying SLPS surface active adsorption sites were equally likely and interaction forces between different phenol molecules did not exist during the adsorption process, regardless of whether the adjacent space had been occupied by other molecules. The separation factor of the Langmuir model was calculated by Equation (6), and the maximum value was 0.67, 0.61, and 0.61 at 20, 30, and 40 ℃, respectively. The separation factor greater than 0 and less than 1 revealed that the adsorption process of phenol onto SLPS benefited the content range investigated [47]. By comparing the adsorption value under three different temperatures, the adsorption efficiency at 30 °C was shown to be better than the others. The poor adsorption efficiency at a low temperature (20 ℃) may be due to the low molecular kinetic energy of phenol, which led to its inactivity in solution. In addition, phenol adsorption is an exothermic reaction, so, the high temperature inhibited the adsorption of phenol by SLPS at 40 °C. The equilibrium data were fitted appropriately with the Langmuir model, and the max. absorbability of SLPS for phenol was around 31.08 mg/g at 30 °C.

## 4. Conclusions

In this study, the water treatment material (SLPS) was successfully synthesized using sodium lignosulfonate modified polystyrene that was functionalized with amine groups. The results indicated that the SLPS could efficiently adsorb phenol in aqueous solution. The adsorption efficacy of SLPS for phenol was related to the initial pH value of solution, adsorption time, temperature, and initial phenol content. The initial pH value of the solution had a substantial influence on the adsorption of SLPS for phenol, and both the acidic and alkaline solutions were not beneficial for phenol adsorption. The pseudo second-order model agreed with the adsorption testing results and was more suitable for representing the kinetic behavior. The adsorption of SLPS for phenol mostly happened in the early phases of the adsorption process and was dominated by chemisorption. Adsorption isotherm analysis showed that the adsorption of SLPS for phenol was well-supported by the Langmuir isotherm model, indicating that the adsorption was monolayer adsorption. The adsorption effect of SLPS for phenol at 30 ℃ was better than at 20 or 40 ℃ and the max. absorbability was 31.08 mg/g. This work provided an effective and novel adsorbent for eliminating phenolic pollutions from aqueous solution and offered an approach for reusing abundant industrial waste.

## Figures and Tables

**Table 1:** Porous structure parameters of samples.

Sample	*S_BET_* (m^2^/g)	*S_mic_* (m^2^/g)	*V_tot_* (cm^3^/g)	*V_mic_* (cm^3^/g)	*D_p_* (nm)
SL	0.38	0.00	0.00	0.00	11.75
CPS	36.21	6.54	0.41	0.01	45.38
SLPS	18.26	0.99	0.30	0.00	66.99

**Table 2:** Adsorption kinetics model parameters of SLPS for phenol.

Model	Parameters	Value
PFO	$q_{e}$	26.407
	$K_{L}$	1.287
	$R^{2}$	0.959
PSO	$q_{e}$	28.963
	$K_{2}$	0.060
	$R^{2}$	0.990
Elovich	*α*	150.639
	β	0.204
	$R^{2}$	0.973

**Table 3:** Adsorption isotherm parameters of SLPS for phenol.

*T* (°C)		Langmuir		Freundlich		
	$q_{m}$ mg/g	$K_{L}$ L/mg	$R^{2}$	$K_{F}$ L/mg	1n L/mg	$R^{2}$
20	33.599	0.010	0.962	3.051	0.368	0.875
30	37.350	0.013	0.967	4.240	0.339	0.892
40	28.241	0.013	0.958	3.308	0.332	0.855
